# How users make judgements about the quality of online health information: a cross-sectional survey study

**DOI:** 10.1186/s12889-022-14418-9

**Published:** 2022-11-01

**Authors:** Wenjing Pian, Laibao Lin, Baiyang Li, Chunxiu Qin, Huizhong Lin

**Affiliations:** 1 School of Economics and Management, Fuzhou University, Fuzhou city, China; 2 Center for Studies of Information Resources, Wuhan University, Wuhan city, China; 3 Laboratory of Data Intelligence and Interdisciplinary Innovation, Nanjing University, Nanjing city, China; 4 Department of Information Management, School of Economics and Management, XIDIAN University, Xi’An city, China; 5 Department of Cardiology, Fujian Medical University Union Hospital, Fuzhou city, China

**Keywords:** Health information, Information quality, Judgement criteria, Social media, eHealth literacy, Infodemic

## Abstract

**Background:**

People increasingly use the Internet to seek health information. However, the overall quality of online health information remains low. This situation is exacerbated by the unprecedented “infodemic”, which has had negative consequences for patients. Therefore, it is important to understand how users make judgements about health information by applying different judgement criteria.

**Objective:**

The objective of this study is to determine how patients apply different criteria in their judgement of the quality of online health information during the pandemic. In particular, we investigate whether there is consistency between the likelihood of using a particular judgement criterion and its perceived importance among different groups of users.

**Methods:**

A cross-sectional survey was conducted in one of the leading hospitals in a coastal province of China with a population of forty million. Combined-strategy sampling was used to balance the randomness and the practicality of the recruiting process. A total of 1063 patients were recruited for this study. Chi-square and Kruskal–Wallis analyses were used to analyse the survey data.

**Results:**

In general, patients make quality judgement of health information more frequently based on whether it is familiar, aesthetic, and with expertise. In comparison, they put more weights on whether health information is secure, trustworthy, and with expertise when determining its quality. Criteria that were considered more important were not always those with a higher likelihood of being used. Patients may not use particular criteria, such as familiarity, identification, and readability, more frequently than others even if they consider them to be more important than other do and vice versa. Surprisingly, patients with a primary school degree put more weight on whether health information is comprehensive than those with higher degrees do in determining its quality. However, they are less likely to use this guideline in practice.

**Conclusions:**

To the best of our knowledge, this is the first study to investigate the consistency between the likelihood of using certain quality judgement criteria and their perceived importance among patients grouped by different demographic variables and eHealth literacy levels. The findings highlight how to improve online health information services and provide fine-grained customization of information for users.

**Supplementary Information:**

The online version contains supplementary material available at 10.1186/s12889-022-14418-9.

## Introduction

Internet services have given users convenient access to various types of online information resources. A worldwide study in 2018 reported that more than 4 billion users searched for information via the internet [1], most of whom searched for health information. More than 70% of U.S. adults were reported to perform such searches [2], as did European adults [3] and Chinese citizens [4]. They use the information to facilitate diagnosis, manage conditions, consult with their doctors and make informed medical decisions for themselves and others [5–9]. However, its overall quality remains low [10, 11]. This situation is exacerbated by the COVID-19 pandemic and the unprecedented "infodemic", which is characterized by a flood of rumours and conspiracy theories through various online platforms [12]. During the pandemic, low-quality information resulted in negative consequences for patients due to anxiety about deteriorating conditions, delayed treatments [13] and even death in extreme cases [14]. The quality of information has been defined in different ways. It has been defined as "fitness for use" [15], which is difficult to apply to the judgement of health information due to its vagueness. It has also been defined as meeting or exceeding consumer expectations [16] with a focus on the value of particular information to consumers and the satisfaction of users’ needs. Personal judgement of its quality is a process that involves the subjective evaluation of information based on the user's own information needs and characteristics [17]. Hence, users may make judgements for different reasons or based on certain criteria (e.g., whether it is objective, comprehensive, and accurate). Therefore, it is important to understand how users use these criteria to make judgements and what factors affect the way they use these criteria.

The objective of this study is to determine how patients apply these different criteria in their judgement of the quality of online health information during the pandemic in terms of how frequently they apply these criteria and how important they consider them. In particular, we investigate whether there is consistency between the likelihood of using a particular criterion and its perceived importance among groups of users with different demographics (age, gender, and educational level) and levels of health literacy.

### Models/frameworks of information quality judgement and their criteria

Different conceptualizations of information quality have been proposed in previous literature. The process-oriented model views information quality as a product of measurement where accuracy is guaranteed during the measurement process [18], while the system-oriented model defines information quality as various steps of information collection, retrieval, and display [19]. In comparison, the user-oriented model conceptualizes information quality as users’ subjective perception of whether information satisfies their information needs [20], and information quality judgement is the process by which users apply quality criteria to determine the overall quality of certain information [20]. This study adopts the user-oriented approach to investigate how users make subjective judgements on the quality of health information.

Several user-oriented models were found in previous literature. Bovee, Srivastava, and Mak (2003) proposed an intuitive model of information quality, proposing a three-layer model with integrity, accessibility, interpretability, and relevance in the first layer. Integrity included four criteria: accuracy, completeness, consistency, and existence. Datedness, usefulness, and other criteria were included as the second layer of the relevance criterion. Age and volatility were included within datedness as the third layer of the criteria [21]. Kahn, Strong, and Wang (2002) developed a two-by-two conceptual model that defined two dimensions of information quality: one dimension for product quality and service quality and the other for conforming to specifications and meeting or exceeding consumer expectations. They assigned multiple criteria to these quadrants, such as freedom from errors, conciseness, completeness, and consistency within the quadrant "product quality * conforms to specifications" and timeless and security within the quadrant "service quality * conforms to specifications" [17]. Stvilia et al. (2007) developed a three-category framework, including intrinsic, relational, and reputational categories. Within each category, several criteria were included (e.g., currency and complexity within the intrinsic category and accuracy and naturalness in the relational category) [22].

Sellitto and Burgess (2005) developed a weighted framework consisting of criteria with different weights [5]. Criteria "reputable" was assigned the highest weight, followed by "no advertising" and "creation”. Stvilia, Mon, and Yi (2009) extended Stivilia's model (2007) and suggested a model of consumer health information quality with five constructs: accuracy, completeness, authority, usefulness, and accessibility, each of which contained several subcriteria (e.g., credibility and reliability in the construct of accuracy) [23]. Subsequently, Kim and Oh (2009) developed a fine-grained list of criteria for information quality judgement, including clarity, rationality, novelty, and quickness [24], which were not included in the list of Stvilia et al. Sun et al. (2015) further extended the list by identifying additional criteria: solution feasibility, certification, politeness and humour [25]. Other lists of judgement criteria have also been found in the works of other scholars (e.g., see Choi & Shah, 2016 [26]; Emamjome et al., 2013 [27]). They share most of their criteria with the aforementioned lists.

The issue with these studies is that terms were used inconsistently within different lists of criteria. For instance, the consistency criterion from Stvilia's study was replaced by the term readability in Zhu et al.'s study with the same definition [28], which was further changed to representation interpretability in Ge et al.'s study [29]. Besides, currency was interchanged with timeliness and quickness in different studies [30]. A summary of the aforementioned criteria is presented in Table 1. Recently, Sun et al. (2019) conducted a systematic review of quality judgement of online health information and summarize all the criteria in previous literature into a list of twenty-five criteria [11]. This comprehensive list of criteria was used in this study.

**Table 1:** Models of information quality judgment and their criteria

Authors	Publication date	List of judgement criteria for informatoin quality
Bovee et al [21]	2003	Integrity, accessibility, Interpretability, and Relevance
Kahn et al [17]	2002	Accessibility, appropriate amount of information, believability, completeness, concise representation, consistent representation, ease of manipulation, free of error, interpretability, objectivity, relevancy, reputation, security, timeless, understandability, and value-added
Sellitto & Brugess [5]	2005	Auhority and currency, accuracy, objectivity, and privacy
Stvilia et al [22]	2007, 2008	Accuracy/validity, cohesiveness, complexity, semantic consistency, structural consistency, currency, informativeness, naturalness, precision, relevance, accessibility, security, and verifiablity
Stvilia et al [23]	2009	Authoritative, complementarity, privacy, attribution, justifiability, transparency, financial disclosure, and advertising policy
Emamjome et al [27]	2013	Completeness, originality, objectivity, novelty, accuracy, content quality, verifiability, reliability, amount of data, relevancy, credibility, user feedback, timeless, understandability, value-added, conciseness, consistency, and accessibility
Weiskopf & Wen	2013	Completeness, correctness, concordance, plausibility, and currency
Sun et al [25]	2015	Accuracy, specificity, objectivity, completeness, relevance, language expression, valuable words, novelty, understandability, profession, originality, external links, external certification, quickness, interactivity, effectiveness, and solution feasibility
Choi & Shah [26]	2016	Responsiveness, alternativeness, completeness, emotional support, verifiability, and trustworthy

### Factors influencing the judgement of health information quality

Several factors have been found to influence the judgement of health information quality. Benotsch, Kalichman, and Weinhardt (2004) conducted a survey of HIV patients to determine how patients evaluated the quality of online health information for certain health websites [18]. Educational and income levels were found to be significantly related to the overall assessment of online health information quality in addition to health literacy (including knowledge) and other psychological factors. Another national survey of Americans’ health information seeking indicated that educational level affected users' ability to navigate within the online environment to seek health information [31], which affected their judgement of online health information.

Another set of studies focused on the factors that are related to particular criteria for the quality of health information, i.e., trustworthiness and credibility. Age, sex, and education were found to be significantly related to these criteria [32]. Three reviews summarized a list of possible influencing factors found in empirical studies: gender, education, health status, income, age, health literacy, race, and health beliefs. However, health status has been found to be significantly related to trustworthiness and credibility in certain studies but not in others [33–37]. In addition, controversial results on health beliefs have been found (e.g., Atkinson et al. found that people with poor health status trusted online health information more, while Cotten et al. observed the opposite [33–35, 38, 39]). Furthermore, health beliefs have been found to affect the intention to use health information rather than the direct judgement of quality [40]. Therefore, these two factors were left for further investigation, while gender, age, education level, and health literacy were retained in this study. In addition, the health literacy scale was replaced with the eHealth literary scale, which is more appropriate for the online environment. Race was not used in this study since the participants were all Chinese.

Although quite a few studies have focused on the overall judgement of health information quality or examined some of its dimensions/criteria while ignoring other criteria, no previous studies have investigated both how likely users are to use certain quality judgement criteria and how important they consider these criteria. In other words, it remains unclear whether the likelihood of using certain criteria is consistent with the perceived importance of these criteria among users with different demographic characteristics. This study investigated how these demographic variables and eHealth literacy affect these patterns and consistency.

### Research methods

#### Research setting and sampling procedure

The research participants were recruited from one of the leading hospitals in a coastal province of China with a population of forty million for the following reasons. First, the hospital is listed in the top 100 hospitals of China^1^ and is the most comprehensive tertiary hospital in that province, with more than two million visits annually. Second, the hospital provides health services to patients from each region of the province, which increases the representativeness of the sample.

Combined-strategy sampling was used to balance the randomness and the practicality of the recruiting process [41]. Given the large number of departments within the hospital, randomization stratified for inpatient and outpatient divisions was undertaken to select the target departments where the patients were recruited. Within each department, a systematic sampling procedure was performed with a randomized seeding number assigned to recruit patients. A paper-based survey was administered, and the data were collected from September to October 2021. The participants (who were 18 years old or above) were approached by nurses in person and briefed with the objective of this study. They were asked to sign an informed consent form before the survey began. In addition, they were able to withdraw from the survey at any time, and anonymity was ensured. Surveys were conducted following confirmation of informed consent which was signed prior to the survey questions. This consent process was approved by the the Institutional Review Board (IRB) of Fujian Medical University Union Hospital (IRB No. 2022KY004). All methods were performed in accordance with the relevant guidelines and regulations (Declaration of Helsinki).

To determine the sample size, two guidelines were considered. According to the COnsensus-based Standards for the selection of health Measurement Instruments (COSMIN) criteria, the ideal sample size for a survey should be no less than ten times the number of survey items [42]^.^ In addition, a power analysis was performed to calculate the minimum sample size for a study with variance analysis of between-group differences. The minimum sample size was 231, with a predefined effect size of 0.40 (which is considered large for six groups), error margin of 0.01, and power of 0.99 for a priori analysis. Therefore, a sample of one thousand participants was considered sufficient for this study (the study included forty survey questions).

### Measure and data analysis

The demographics of the participants were collected and categorized as follows. In terms of age, the participants were categorized into five groups from 18 years old to 60 years and above (18–29; 30–39; 40–49; 50–59; 60 and above). Educational levels were also categorized into five groups from junior high school or below to Ph.D. degree (junior high school or below; high school; undergraduate; master’s; Ph.D.). When gender was considered, two groups (female; male) were used to categorize the participants (no participant indicated gender as unclear or transgender).

In addition to the demographic variables, two sets of questions were used in this study. The first set was items to assess the intention of using a particular criterion in the judgement of online health information and the perceived importance of that criterion, adopted from Sun et al.'s review article [11]. For each criterion, the participant was asked to indicate whether he or she used it in evaluating the quality of online health information. If yes, the participants proceeded to indicate how important the criterion was using a five-point Likert scale. The second set was the eHealth Literacy scale (eHEALS) to assess consumers’ ability to engage in eHealth [43]. It was used to replace the traditional health literacy scales since the context of this study was online health information seeking and evaluation. This scale has been validated by various empirical studies and shows good psychometric properties. The distribution of the eHealth literacy scores among the participants was skewed (S = -0.446), with a median total score of 30 for the eHEALS scale (range: 8–40). Since there was no consensus on the cut-off score of the eHEALS scale to categorize people into high and low eHealth literacy, we used the median score to divide the participants into these two user groups.

All the measures used in the study were forwards and backwards translated following the COSMIN guidelines. Each type of translation involved two bilingual translators whose mother tongue was the original language: one was the domain expert, and the other was naive about the domain. The backwards translation was compared with the original questions, and discrepancies were resolved by discussion among the authors and translators.

Two kinds of statistical analyses were adopted in this study. To determine whether a particular criterion was used by a patient, chi-square analysis was conducted to compare the group differences in the frequency of criterion use since it was a binary variable. For the perceived importance of each criterion, nonparametric analysis (Kruskal–Wallis test and 1-way ANOVA for post hoc test) was used to compare the group differences (the normal distribution assumption was not guaranteed). It should be noted that in the analysis of importance, participants who indicated that they did not use a particular criterion were excluded since the measurement of the five-point Likert scale assumed that the participants used the criterion regardless of how important they considered it (even if it was considered unimportant, the value was 1 in the scale). Participants who did not use the criterion would introduce bias into the analysis interpretation of the results. The scores of the eHealth literacy scale items were summed to indicate participants’ level of literacy when they conducted online health information seeking and evaluation [44]. The data analysis was conducted using SPSS 26 software.

## Results

### Descriptive results of participants' demographics

A total of 1500 patients were invited to participate in this study, of whom 1063 accepted the invitation and finished the survey (acceptance rate: 70.9%). Among them, nearly two-thirds were under forty years old, while less than 10% were over sixty. In terms of educational level, more than half of the participants held a college degree, and more than one-quarter of them had received higher degrees (master’s and Ph.D.). In comparison, less than 20% held a primary or high school degree. Details are shown in Table 2.

**Table 2:** Demographics of research participants

Demographic variable		Frequency	Percentage
Education	Junior high school and below	27	2.5
	High school	153	14.4
	Undergraduate	604	56.8
	Master	275	25.9
	PhD	4	0.4
Gender	Male	411	38.7
	Female	652	61.3
Age	18–29	477	44.9
	30–39	240	22.6
	40–49	149	14.0
	50–59	102	9.6
	60 and above	95	8.9
		**Mean(Standard Deviation)**	**Skewness(Standard Error)**
eHealth Literacy		3.7399(0.439)	-0.446(0.075)

Regarding the eHealth literacy level of the participants, the distribution was skewed (S = -0.446) with a median total score of 30 for the eHEALS scale (range: 8–40). This is similar to recent survey results of eHealth literacy from other countries using the same scale [45, 46]. Since there was no consensus on the cut-off score of the eHEALS scale to categorize people into high and low eHealth literacy, we used the median score to divide the participants into these two user groups.

### The frequency of the use of judgement criteria on health information quality and the perceived importance among different user groups

The statistical results of the frequency of use for the judgement criteria for health information quality among different user groups and the perceived importance are presented according to the demographic variables and eHealth literacy levels mentioned above. The details of the post hoc analysis can be found in the appendix.

### eHealth literacy

Patients with high eHealth literacy were more inclined to use the criteria of transparency and usefulness than those with low eHealth literacy. However, there were no differences in the perceived importance of these two criteria between these two groups of patients. In contrast, for the criteria of familiarity, accessibility, aesthetics, comprehensiveness, and practicality, no significant differences were found in their frequencies of use between the two groups of participants, although they perceived them to have significantly different importance. The nonparametric statistical analysis (K-W) showed that patients with high eHealth literacy considered these criteria to be more important than did those with low eHealth literacy. For the criteria of identification, interactivity and balance, patients with high eHealth literacy considered these criteria to be more important and therefore were more inclined to use them than those with low eHealth literacy. No differences in the frequency of use and perceived importance were found for the remaining criteria between patients with high and low eHealth literacy. Details are shown in Table 3.

**Table 3:** Chi-square and K-W tests for patients with different eHealth literacy levels

	**Chi-Square test (for frequency of use)**			**Kruskal–Wallis test (for perceived importance)**		
	Value	df	Asymp.Sig	χ^2^	df	Asymp.Sig
Trustworthiness	2.525	1	0.112	0.446	1	0.504
Expertise	3.783	1	0.052	0.833	1	0.362
Objectivity	1.888	1	0.169	0.093	1	0.761
Transparency	4.323	1	0.038^a^	0.001	1	0.97
Popularity	1.116	1	0.291	0.989	1	0.32
Understandability	1.888	1	0.169	0.015	1	0.903
Relevance	2.147	1	0.143	2.15	1	0.143
Familiarity	0.087	1	0.768	8.358	1	0.004
Accessibility	1.888	1	0.169	6.499	1	0.011^a^
Identification	4.725	1	0.030^a^	5.779	1	0.016^a^
Believability	1.888	1	0.169	1.085	1	0.298
Accuracy	3.393	1	0.065	0.066	1	0.798
Readability	1.116	1	0.291	0.264	1	0.607
Currency	1.008	1	0.315	0.001	1	0.973
Navigability	3.138	1	0.076	2.807	1	0.094
Aesthetics	3.783	1	0.052	18.345	1	0.000^c^
Interactivity	6.324	1	0.012^a^	12.881	1	0.000^c^
Comprehensiveness	2.363	1	0.124	13.257	1	0.000^c^
Practicality	3.138	1	0.076	6.757	1	0.009^b^
Completeness	1.116	1	0.291	0.624	1	0.43
Usefulness	9.914	1	0.002^b^	1.311	1	0.252
Balanced	8.464	1	0.004^b^	4.01	1	0.045^a^
Anonymity	2.998	1	0.083	1.51	1	0.219
Security	1.045	1	0.307	0.197	1	0.657
Learnability	3.715	1	0.054	0.568	1	0.451

^a^indicates the significance level < 0.05

^b^< 0.01

^c^< 0.001

### Age

For the criteria of familiarity, identification, aesthetics, and anonymity, no significant differences were found in the likelihood of using these criteria in judging the quality of online health information among different age groups. However, these criteria were considered to have different levels of importance among these groups. The nonparametric statistical analysis (K-W) showed that for the criteria of familiarity, identification, and aesthetics, patients from 18 to 29 years old considered them less important than did those from 30 to 39, 40 to 49, and 50–59 years old. In comparison, the difference between any other two age groups was not significant. In terms of the criterion of anonymity, only patients from 18 to 29 years old considered it less important than those from 40 to 49 years old did. Surprisingly, patients over 60 years old were least likely to use this criterion. Patients from 30 to 39 years old were found to have the second-lowest likelihood of adopting this criterion. In addition, patients from 18 to 29 years old were less likely than those aged 40 to 59 years old to apply this criterion to the quality judgement of online health information. For the other criteria, no significant differences were found in either the likelihood of use or the perceived importance among patients in different age groups. Details are shown in Table 4.

**Table 4:** Chi-square and K-W tests for patients in different age groups

	**Chi-Square test (for frequency of use)**			**Kruskal–Wallis test (for perceived importance)**		
	Value	df	Asymp.Sig	χ^2^	df	Asymp.Sig
Trustworthiness	1.971	4	0.741	4.336	4	0.362
Expertise	2.173	4	0.704	5.327	4	0.255
Objectivity	1.881	4	0.758	1.028	4	0.906
Transparency	1.093	4	0.895	2.645	4	0.619
Popularity	4.299	4	0.367	2.931	4	0.57
Understandability	15.218	4	0.004^b^	7.74	4	0.102
Relevance	4.246	4	0.374	6.033	4	0.197
Familiarity	4.092	4	0.394	13.377	4	0.01^a^
Accessibility	0.125	4	0.998	7.387	4	0.117
Identification	1.370	4	0.849	14.91	4	0.005^b^
Believability	3.369	4	0.498	4.798	4	0.309
Accuracy	0.801	4	0.938	4.237	4	0.375
Readability	4.525	4	0.340	4.027	4	0.402
Currency	3.549	4	0.470	3.395	4	0.494
Navigability	1.609	4	0.807	6.025	4	0.197
Aesthetics	2.173	4	0.704	33.824	4	0.000^c^
Interactivity	2.250	4	0.690	2.075	4	0.722
Comprehensiveness	2.603	4	0.626	2.827	4	0.587
Practicality	0.969	4	0.914	1.232	4	0.873
Completeness	4.299	4	0.367	4.503	4	0.342
Usefulness	2.196	4	0.700	4.132	4	0.388
Balanced	7.756	4	0.101	4.249	4	0.373
Anonymity	3.623	4	0.459	12.744	4	0.013^a^
Security	2.840	4	0.585	3.957	4	0.412
Learnability	4.655	4	0.325	1.147	4	0.887

^a^indicates the significance level < 0.05

^b^< 0.01

^c^< 0.001

### Educational level

A similar situation was found among patients with different educational levels. For the criteria of expertise, familiarity, aesthetics, and security, patients with different educational levels were found to have no significant differences in their inclination to use these criteria. However, they assigned different importance to these criteria. The expertise criterion was considered more important by patients with a master's degree than by patients with either a bachelor's or a high school degree. Similarly, the security criterion was considered more important by patients with a master's degree than by those with either a bachelor's or a high school degree. In contrast, familiarity was considered more important by patients with either a bachelor's or high school degree than by those with a master's degree. Furthermore, patients with high school degrees considered familiarity even more important than those with a bachelor's degree. For the criteria of objectivity, identification, and accuracy, no significant differences in their perceived importance were found among patients with different educational levels. However, their likelihood of being used differed significantly among these patient groups. For the objectivity criterion, patients with primary school degrees were more inclined to use this criterion than those with higher degrees when making a quality judgement about online health information. Surprisingly, patients with a Ph.D. degree were more inclined to ignore this criterion than those with either a bachelor's or a master's degree. It should be noted that patients with a primary school degree were less likely to use this criterion for quality judgement than patients with any other educational level. In sharp contrast, they considered this criterion to be more important than those with a bachelor's degree. Details are shown in Table 5.

**Table 5:** Chi-square and K-W tests for patients with different educational levels

	**Chi-Square test (for frequency of use)**			**Kruskal–Wallis test (for perceived importance)**		
	Value	df	Asymp.Sig	χ^2^	df	Asymp.Sig
Trustworthiness	4.042	4	0.400	9.303	4	0.054
Expertise	5.032	4	0.284	18.212	4	0.001^b^
Objectivity	15.192	4	0.004^b^	6.544	4	0.162
Transparency	1.949	4	0.745	4.601	4	0.331
Popularity	4.432	4	0.351	8.07	4	0.089
Understandability	7.272	4	0.122	1.466	4	0.833
Relevance	6.661	4	0.155	3.647	4	0.456
Familiarity	5.032	4	0.284	12.408	4	0.015^a^
Accessibility	3.337	4	0.503	2.555	4	0.635
Identification	11.073	4	0.026^a^	6.133	4	0.189
Believability	2.777	4	0.596	3.863	4	0.425
Accuracy	10.340	4	0.035^a^	5.299	4	0.258
Readability	5.753	4	0.218	3.194	4	0.526
Currency	3.095	4	0.542	5.937	4	0.204
Navigability	5.753	4	0.218	1.809	4	0.771
Aesthetics	6.449	4	0.168	14.454	4	0.006^b^
Interactivity	8.492	4	0.075	5.685	4	0.224
Comprehensiveness	22.505	4	0.000^c^	11.543	4	0.021^a^
Practicality	5.940	4	0.204	2.978	4	0.562
Completeness	4.432	4	0.351	0.44	4	0.979
Usefulness	3.331	4	0.504	1.765	4	0.779
Balanced	6.389	4	0.172	1.321	4	0.858
Anonymity	2.289	4	0.683	0.724	4	0.948
Security	2.538	4	0.638	12.353	4	0.015^a^
Learnability	11.227	4	0.024^a^	2.058	4	0.725

^a^indicates the significance level < 0.05

^b^< 0.01

^c^< 0.001

### Gender

For the criteria of trustworthiness, relevance, believability, readability, practicality, completeness, anonymity, and security, female patients considered them to be more important than male patients did. However, female patients were not more inclined to use these criteria than male patients in judging the quality of online health information. No significant differences in the likelihood of using the remaining criteria and their perceived importance were found between these two groups of patients. Details are shown in Table 6.

**Table 6:** Chi-square and K-W tests for patients in different genders

	**Chi-Square test (for frequency of use)**			**Kruskal–Wallis test (for perceived importance)**		
	Value	df	Asymp.Sig	χ^2^	df	Asymp.Sig
Trustworthiness	0.956	1	0.328	9.308	1	0.002^b^
Expertise	0.072	1	0.788	2.745	1	0.098
Objectivity	1.035	1	0.309	1.928	1	0.165
Transparency	0.052	1	0.820	3.403	1	0.065
Popularity	0.437	1	0.509	0.044	1	0.834
Understandability	0.128	1	0.721	0.296	1	0.586
Relevance	1.015	1	0.314	4.485	1	0.034^a^
Familiarity	0.072	1	0.788	1.492	1	0.222
Accessibility	0.109	1	0.742	3.039	1	0.081
Identification	0.145	1	0.703	1.239	1	0.266
Believability	1.092	1	0.296	14.725	1	0.000^c^
Accuracy	0.000	1	0.988	2.101	1	0.147
Readability	0.005	1	0.946	4.767	1	0.029^a^
Currency	0.658	1	0.417	3.626	1	0.057
Navigability	0.437	1	0.509	2.179	1	0.14
Aesthetics	0.327	1	0.567	0.297	1	0.586
Interactivity	0.377	1	0.539	1.235	1	0.266
Comprehensiveness	1.777	1	0.183	0.333	1	0.564
Practicality	0.635	1	0.426	7.886	1	0.005^b^
Completeness	0.635	1	0.426	4.418	1	0.036^a^
Usefulness	0.025	1	0.875	0.006	1	0.937
Balanced	0.915	1	0.339	0.606	1	0.436
Anonymity	0.046	1	0.830	5.423	1	0.02^a^
Security	0.008	1	0.931	3.982	1	0.046^a^

^a^indicates the significance level < 0.05

^b^< 0.01

^c^< 0.001

## Discussion

To our knowledge, this is the first study to investigate the consistency between the likelihood of using particular quality judgement criteria and their perceived importance among different groups of patients when making quality judgements about online health information. It was found that for particular criteria such as familiarity, identification, and readability, patients in one demographic group may not use them more frequently than other groups even if they consider these criteria more important than the other groups do, while patients in particular groups may use these criteria more frequently even if they do not consider them more important than other groups do. Furthermore, it is surprising that patients with a primary school degree considered the comprehensiveness criterion to be more important than those with a bachelor's degree but were less likely to use it in practice, which is counterintuitive to common sense. In the next sections, the results are interpreted and discussed with both theoretical and practical implications.

### Theoretical implications

The inconsistency between the likelihood of use and perceived importance indicates that patients may use different sets of quality judgement criteria in different ways. The criteria of popularity, currency, and navigability are those in which no significant difference was found in either the likelihood of use or perceived importance among the patients grouped by all the demographic variables and eHealth literacy. This suggests that these criteria may be independent of patients’ demographic and eHealth literacy backgrounds. Patients share similar attitudes regarding their importance and how to use them. The underlying reason could be that applying these criteria to the quality judgement of online health information does not involve patients' knowledge, experience, and other cognitive features, which are influenced by their age, education, health literacy, and gender. Another possible reason could be that there are universal prototypes for popularity, currency, and navigability among all patients and even the entire population. Therefore, demographic variables may not affect whether these criteria are used and how important they are perceived to be. Unfortunately, no previous studies were found in this area, and further investigation is required to test these possible explanations.

In contrast, the perceived importance of criteria does not always guarantee the inclination to use these criteria. For example, patients with a master's degree assigned more weight to the expertise criterion than those with either a high school or bachelor's degree. However, in practice, they did not show a preference for this particular criterion over the other two groups of patients. The reason could be that the more education patients receive, the more attention they give to features that are closely related to the information content. Hence, the perception of expertise and security conveyed by the information is more attractive to well-educated patients. In comparison, for the criterion of aesthetics, the opposite was observed. The same reasoning could be applied to the perceived importance of the criterion of familiarity among different age groups. As patients get older, they may have fewer cognitive resources and may rely on more superficial features of online health information, such as the heuristic of familiarity, to assess its overall quality.

It should be noted that for the criterion of comprehensiveness, although patients with a primary school degree considered it more important than those with a bachelor’s degree, they were actually more likely to ignore it when making a quality assessment of online health information. It is common sense that if one group of people considers particular criteria more important than another group, they tend to use these criteria more frequently. This counterintuitive finding may be due to the irrational reasoning and decision-making of these patients during a crisis (i.e., the COVID-19 pandemic and infodemic). During the infodemic, patients are likely to be influenced by the risk situation and emotion [47, 48], which further biases their rational thinking process and affects their quality judgement. Therefore, they may not naturally rely on the criteria they consider important [49], which contributes to the inconsistency.

### Practical implications

The findings of this study provide guidelines on how online health information should be presented to facilitate users' quality judgement, which further helps them use information appropriately.

First, online health information services require fine-grained customization to facilitate the evaluation of users with different demographic characteristics. As patients can be grouped into fine-grained user groups by combining these demographic variables, this study provides practical guidelines on prioritizing detailed user groups during the customization of online health information services. There are different priority sequences for these user groups to facilitate their quality judgement.

Second, the health literacy of patients should be given more attention when attempting to motivate patients to find higher-quality health information. As one of the main drivers of health behavioural change [50], this may influence patients’ willingness to follow certain online suggestions and perform particular types of protective behaviours, even if these suggestions are of low quality. Therefore, it is necessary for scholars to develop more efficient interventions to increase patients’ health literacy.

### Research limitations and directions for future research

Several limitations deserve attention when interpreting the results of this study. First, only one of the leading hospitals of the coastal province with a population of forty million was chosen as the research context for this study. Hence, patients from other hospitals in this coastal province were not included in this study, nor were patients from different provinces of the country. However, this hospital was listed in the top 100 hospitals of China and was the top hospital with more than two million visits annually. Second, the number of patients who did not use a particular criterion in their judgement was small, which may bias the statistical analysis and validity of the results. Third, income level and other demographic variables were not explored in this study. The participants from this study considered their income sensitive and private information and refused to provide this information even though they were ensured that the information would be confidential. Fourth, this study did not take other confounding variables into consideration, which might influence the interpretation of the results. This could be further explored in future studies. Fifth, only patients from China were included in this study. It is possible that samples from other countries may exhibit different patterns, which could be further investigated in the future.

Future research could be conducted in the following two directions. One direction would be to further explore the reasons for the inconsistency between the likelihood of using specific quality judgement criteria for online health information and their perceived importance. Although several possible explanations were proposed in this study, the underlying mechanisms remain unclear. The other direction is to further investigate the influence of other variables (e.g., different types of health information needs and people’s resilience) [8, 51–53] on the use patterns of quality judgement criteria to provide a more comprehensive understanding of this topic.

## Conclusion

To the best of our knowledge, this is the first study to investigate the consistency between the likelihood of using certain quality judgement criteria and their perceived importance among patients grouped by different demographic variables and eHealth literacy levels. It was found that the patterns are not always consistent among patients of different ages, genders, educational levels and eHealth literacy levels. The perceived greater importance of particular criteria by certain patient groups does not guarantee a higher likelihood of using these criteria in practice and vice versa. The criterion of comprehensiveness was perceived as more important by patients with a primary school degree than by those with a bachelor's degree, although it was less likely to be used. Possible reasons for these findings include the different nature of these criteria and the existence of stable prototypes for a certain set of criteria. The findings highlight how to improve online health information services and provide fine-grained customization of information for users to make judgements easier and faster.

## Supplementary Information

**Additional file 1. **Appendix.

## Data Availability

The datasets generated and/or analysed during the current study are not publicly available due ethical permissions not allowing data sharing, but they are available from the corresponding author on reasonable request.

## Footnotes

1 https://www.cn-healthcare.com/articlewm/20220721/content-1404153.html
