# Supplementary material for: How users make judgements about the quality of online health information: a cross-sectional survey study
Source: BMC Public Health. 2022 Nov 1;22:2001. doi: 10.1186/s12889-022-14418-9 (PMC9628111; doi:10.1186/s12889-022-14418-9)
Supplement: Supplementary file 1 — Additional file 1. Appendix. [file 12889_2022_14418_MOESM1_ESM.docx]

## Appendix

1. Chi-Square Test Crosstab for Age

|  |  |  | 18-29 | 30-39 | 40-49 | 50-59 | >=60 | Total |
| --- | --- | --- | --- | --- | --- | --- | --- | --- |
|  |  |  |  |  |  |  |  |  |
| **Understandability** | Use | count |  |  |  |  |  |  |
|  |  |  | 474_a_ | 238_a, b_ | 149_a, b_ | 102_a, b_ | 91_b_ | 1054 |
|  | Do not use | count |  |  |  |  |  |  |
|  |  |  | 3_a_ | 2_a, b_ | 0_a, b_ | 0_a, b_ | 4_b_ | 9 |

| Each subscript indicates a subset of Age categories, and at the .05 level, the column proportions of these categories do not differ significantly from one another. |
| --- |

2. Chi-Square Test Crosstab for Education

|  |  |  | Junior high school and below | High school | Undergraduate | Master | PhD | Total |
| --- | --- | --- | --- | --- | --- | --- | --- | --- |
|  |  |  |  |  |  |  |  |  |
| **Objectivity** | Use | count |  |  |  |  |  |  |
|  |  |  | 25_a_ | 151_a, b_ | 601_b_ | 273_b_ | 4_a, b_ | 1054 |
|  | Do not use | count |  |  |  |  |  |  |
|  |  |  | 2_a_ | 2_a, b_ | 3_b_ | 2_b_ | 0_a, b_ | 9 |
| **Identification** | Use | count |  |  |  |  |  |  |
|  |  |  | 25_a_ | 151_a, b_ | 597_a, b_ | 274_b_ | 4_a, b_ | 1051 |
|  | Do not use | count |  |  |  |  |  |  |
|  |  |  | 2_a_ | 2_a, b_ | 7_a, b_ | 1_b_ | 0_a, b_ | 12 |
| **Accuracy** | Use | count |  |  |  |  |  |  |
|  |  |  | 25_a_ | 151_a, b_ | 596_a, b_ | 274_b_ | 4_a, b_ | 1050 |
|  | Do not use | count |  |  |  |  |  |  |
|  |  |  | 2_a_ | 2_a, b_ | 8_a, b_ | 1_b_ | 0_a, b_ | 13 |
| **Comprehensiveness** | Use | count |  |  |  |  |  |  |
|  |  |  | 24_a_ | 151_b_ | 600_b_ | 270_b_ | 4_a, b_ | 1049 |
|  | Do not use | count |  |  |  |  |  |  |
|  |  |  | 3_a_ | 2_b_ | 4_b_ | 5_b_ | 0_a, b_ | 14 |
| **Learnability** | Use | count |  |  |  |  |  |  |
|  |  |  | 25_a_ | 152_a, b_ | 599_b_ | 272_a, b_ | 4_a, b_ | 1052 |
|  | Do not use | count |  |  |  |  |  |  |
|  |  |  | 2_a_ | 1_a, b_ | 5_b_ | 3_a, b_ | 0_a, b_ | 11 |

| Each subscript indicates a subset of Education categories, and at the .05 level, the column proportions of these categories do not differ significantly from one another. |
| --- |

3. Post hoc test (1-way ANOVA) of the K-W test for Age (Each node shows the sample average rank of Age)

|  | Sample 1-Sample 2 | Test  Statistic | Std.  Error | Std. Test Statistic | Sig. | Adj. Sig |
| --- | --- | --- | --- | --- | --- | --- |
|  |  |  |  |  |  |  |
| **Familiarity** | 18-30-40-50 |  |  |  |  |  |
|  |  | -58.976 | 24.541 | -2.403 | .016* | .163 |
|  | 18-30-30-40 |  |  |  |  |  |
|  |  | -59.860 | 20.428 | -2.930 | .003** | .034* |
|  | 18-30-50-60 |  |  |  |  |  |
|  |  | -61.701 | 28.331 | -2.178 | .029* | .294 |
| **Identification** | 18-30-40-50 |  |  |  |  |  |
|  |  | -58.535 | 24.258 | -2.413 | .016* | .158 |
|  | 18-30-50-60 |  |  |  |  |  |
|  |  | -61.822 | 28.004 | -2.208 | .027* | .273 |
|  | 18-30-30-40 |  |  |  |  |  |
|  |  | -64.716 | 20.192 | -3.205 | .001** | .014* |
| **Aesthetics** | 18-30-40-50 |  |  |  |  |  |
|  |  | -65.337 | 25.948 | -2.518 | .012* | .118 |
|  | 18-30- over 60 years old |  |  |  |  |  |
|  |  | -97.321 | 30.930 | -3.147 | .002** | .017* |
|  | 18-30-50-60 |  |  |  |  |  |
|  |  | -103.537 | 29.955 | -3.456 | .001** | .005** |
|  | 18-30-30-40 |  |  |  |  |  |
|  |  | -108.432 | 21.599 | -5.020 | .000*** | .000*** |
| **Anonymity** | 18-30-40-50 |  |  |  |  |  |
|  |  | -85.077 | 25.137 | -3.385 | .001** | .007** |

Each row tests the null hypothesis that the Sample 1 and Sample 2 distributions are the same.

Asymptotic significances (2-sided tests) are displayed. The significance level is .05.

4. Post hoc test (1-way ANOVA) of the K-W test for educational levels (Each node shows the sample average rank of educational level)

|  | Sample 1-  Sample 2 | Test  Statistic | Std.  Error | Std. Test Statistic | Sig. | Adj. Sig |
| --- | --- | --- | --- | --- | --- | --- |
|  |  |  |  |  |  |  |
| **Expertise** | Undergraduate-Master |  |  |  |  |  |
|  |  | -73.027 | 18.488 | -3.950 | .000*** | .001** |
|  | High school-Master |  |  |  |  |  |
|  |  | -72.051 | 25.670 | -2.807 | .005** | .050* |
| **Familiarity** | Master-  Undergraduate |  |  |  |  |  |
|  |  | 38.264 | 18.906 | 2.024 | .043* | .430 |
|  | Master-  High school |  |  |  |  |  |
|  |  | 89.973 | 26.250 | 3.427 | .001** | .006** |
|  | Undergraduate-High school |  |  |  |  |  |
|  |  | 51.709 | 23.544 | 2.196 | .028* | .281 |
| **Aesthetics** | Master-  Undergraduate |  |  |  |  |  |
|  |  | 40.355 | 19.990 | 2.019 | .044* | .435 |
|  | Master-  High school |  |  |  |  |  |
|  |  | 77.914 | 27.755 | 2.807 | .005** | .050* |
|  | Master-Junior high school and below |  |  |  |  |  |
|  |  | 161.418 | 57.354 | 2.814 | .005** | .049* |
|  | Undergraduate-  Junior high school and below |  |  |  |  |  |
|  |  | 121.062 | 56.025 | 2.161 | .031* | .307 |
| **Comprehensiveness** | Undergraduate-High school |  |  |  |  |  |
|  |  | 60.217 | 23.918 | 2.518 | .012* | .118 |
|  | Undergraduate-  Junior high school and below |  |  |  |  |  |
|  |  | 111.133 | 53.830 | 2.065 | .039* | .390 |
| **Security** | High school-  Master |  |  |  |  |  |
|  |  | -74.824 | 26.898 | -2.782 | .005** | .054 |
|  | Undergraduate-Master |  |  |  |  |  |
|  |  | -59.544 | 19.372 | -3.074 | .002** | .021* |

Each row tests the null hypothesis that the Sample 1 and Sample 2 distributions are the same.

Asymptotic significances (2-sided tests) are displayed. The significance level is .05.
